# *Mab_3083c* Is a Homologue of RNase J and Plays a Role in Colony Morphotype, Aggregation, and Sliding Motility of *Mycobacterium abscessus*

**DOI:** 10.3390/microorganisms9040676

**Published:** 2021-03-25

**Authors:** Ting-Yu Liu, Sheng-Hui Tsai, Jenn-Wei Chen, Yu-Ching Wang, Shiau-Ting Hu, Yih-Yuan Chen

**Affiliations:** 1 Institute of Microbiology and Immunology, School of Life Science, National Yang-Ming University, Taipei 112, Taiwan; drjiks2009@hotmail.com (T.-Y.L.); u901633@gmail.com (S.-H.T.); 2 Department of Microbiology and Immunology, College of Medicine, National Cheng-Kung University, Tainan 701, Taiwan; jc923@mail.ncku.edu.tw; 3 Institute of Basic Medical Sciences, College of Medicine, National Cheng-Kung University, Tainan 701, Taiwan; 4 Department of Biochemical Science and Technology, National Chiayi Univeristy, Chiayi City 600, Taiwan; S1090496@mail.ncyu.edu.tw

**Keywords:** *Mycobacterium abscessus*, *Mab_3083c*, RNase J, colony morphotype, aggregation, sliding motility

## Abstract

*Mycobacterium abscessus* is an opportunistic pathogen causing human diseases, especially in immunocompromised patients. *M. abscessus* strains with a rough morphotype are more virulent than those with a smooth morphotype. Morphotype switch may occur during a clinical infection. To investigate the genes involved in colony morphotype switching, we performed transposon mutagenesis in a rough clinical strain of *M. abscessus*. A morphotype switching mutant (smooth) named *mab_3083c*::Tn was obtained. This mutant was found to have a lower aggregative ability and a higher sliding motility than the wild type strain. However, its glycopeptidolipid (GPL) content remained the same as those of the wild type. Complementation of the mutant with a functional *mab_3083c* gene reverted its morphotype back to rough, indicating that *mab_3083c* is associated with colony morphology of *M. abscessus*. Bioinformatic analyses showed that *mab_3083c* has a 75.4% identity in amino acid sequence with the well-characterized ribonuclease J (RNase J) of *M. smegmatis* (RNase J_Msmeg_). Complementation of the mutant with the RNase J gene of *M. smegmatis* also switched its colony morphology from smooth back to rough. These results suggest that Mab_3083c is a homologue of RNase J and involved in regulating *M. abscessus* colony morphotype switching.

## 1. Introduction

*Mycobacterium abscessus* is a rapid-growing nontuberculous *Mycobacterium* (NTM) and is often present in soil and water [1,2]. It has been found to infect lung, skin, and soft tissue, especially in immunocompromised patients [3,4,5,6] and is the second most common bacterial species isolated from cystic fibrosis patients [7,8]. The prevalence of *M. abscessus* infection is increasing in hospitals in Taiwan [9,10]. Because *M. abscessus* is highly resistant to multiple antibiotics, treatment of *M. abscessus* infections is often difficult [11,12]. It has been shown that *M. abscessus* is resistant to first-line anti-tuberculosis agents such as rifampin and ethambutol [13,14]. Currently, treatment for *M. abscessus* infection consists of intravenous amikacin combined with oral macrolide such as clarithromycin, as suggested by the American Thoracic Society and the Infectious Diseases Society of America [4]. However, the cure rate of *M. abscessus* pulmonary disease is only 30–50%, much lower than other NTM diseases [15]. Fatal *M. abscessus* infections have also been reported, especially after lung transplantation [16,17]. Several reports have indicated that *M. abscessus* can infect both immunodeficient and immunocompetent patients [2,6,18]. 

*M. abscessus* exhibits two different colony morphotypes, smooth and rough [19], with different properties such as cell wall content, hydrophobicity, sliding motility, and aggregation ability [20,21,22]. The rough strain is more virulent than the smooth strain, and the change from smooth to rough morphotype may occur during infection [23,24]. Studies have shown that a difference between the smooth and rough variants is the amount of glycopeptidolipid (GPL), which is a major surface lipid component of *M. abscessus*, *M. smegmatis*, *M. chelonae*, and *M. avium* complex [25,26]. Single nucleotide polymorphisms or multiple indels with GPL locus were identified by several comparative genomics analysis in the isogenic smooth and rough *M. abscessus* pairs [26]. Deficiency in GPL is associated with the rough morphotype and increased invasiveness [21]. Rough morphotype variants are also more potent than smooth variants in stimulating human macrophages through TLR2 to produce cytokines [27]. Cells of rough strains have been shown to persist and multiply in mouse and human monocytes, while those of smooth strains are rapidly cleared [24,26]. 

In order to investigate the mechanism of colony morphotype switch, we attempted to identify genes involved in colony morphology and virulence of *M. abscessus*. In our previous study [28], a transposon mutagenesis was performed in the rough *M. abscessus* cs1c-R strain. A mutant with a smooth morphotype designated *mab_3083c*::Tn was isolated. Results of bioinformatic analyses suggest that *mab_3083c* is a homologue of ribonuclease J. RNase J is an enzyme present in many bacteria and plays an important role in all aspects of mRNA metabolism including ribosomal RNA maturation, mRNA decay and stability [2]. In this study, we investigated the role of the *mab_3083c* gene, homologue of RNase J, in colony morphology switching and surface-associated properties of *M. abscessus*.

## 2. Materials and Methods

### 2.1. Bacterial Strains and Culture Condition

*M. smegmatis* mc^2^155 was purchased from the American Type Culture Collection (ATCC), and the clinical strain *M. abscessus* cs1c was obtained from the Veterans General Hospital, Taichung, Taiwan. *M. abscessus* cs1c-R was a spontaneous mutant derived from strain cs1c [28]. Cells of *M. abscessus* and *M. smegmatis* were grown at 37 °C on Middlebrook 7H11 agar (Difco, Franklin Lakes, NJ, USA) containing 10% OADC (Oleic acid-bovine albumin-dextrose-catalase) (Becton Dickinson, Holdrege, NE, USA) or in 7H9 broth (Difco, Franklin Lakes, NJ, USA) with 10% OADC and appropriate concentration of Tween 80. When required, selective media were prepared with 7H11 agar supplemented with 10% OADC and apramycin (50 μg/mL) or kanamycin (50 μg/mL).

### 2.2. Transposon Mutagenesis

The mutant library made by EZ-Tn5^TM^<KAN-2>Tnp Transposome^TM^ Kit was carried out according to the manufacturer’s instructions (Epicentre, Madison, WI, USA), and the Tn5 insertion site in the target mutant was identified by inverted PCR with primers KAN-2 FP-1 (5′-ACCTACAACAAAGCTCTCATCAACC-3′) and KAN-2 RP-1 (5’-GCAATGTAACATCAGAGATTTTGAG-3′).

### 2.3. Cloning of Mab_3083c and Msmeg_2685 and Creation of Mab_3083c_D89K,H90A_ Mutant

The *mab_3083c* was amplified by PCR using primers *mab_3083c*-F-BglII (5′-TA**AGATCT**GTGGCAGTTACCCCACCC-3′) and *mab_3083c*-R-NheI (5′-AT**GCTAGC**CTAGTCGACGGTGAGCAC-3′), and that of *msmeg_2685* was amplified with primers *msmeg_2685*-F-Acc65I (5′-TG**GGTACC**ATGAGCGCCGAACTCGCG-3′) and *msmeg_2685*-R-NheI (5′-AT**GCTAGC**TCAGATCTCTATGACGGTC-3′) containing restriction enzyme sites (bold-faced). The amplified products were then cloned into the vector pYUB412A [28], generating pYUB_*mab_3083c*. The *mab_3083c_D89K,H90A_* mutations were generated by site-directed mutagenesis with a three-step PCR as described previously [29]. This three-step PCR was performed with primers *mab_3083c*-mut-F (5′-ATAGGATCGCATCACCATCACCATC-3′), *mab_3083c*-mut-R (5′-ATAAGACCAGACGCTCGCCAAC-3′), *mab_3083c*-mut-Fm2 (5′-GCATGAG**AAAGCC**ATCGGGGCCATTCCGTTCCTGCTGAAG-3′), and *mab_3083c*-mut-Rm2 (5′-CTTCAGCAGGAACGGAATGGCCCCGAT**GGCTTT**CTCATGC-3′) containing mutated sequence (bold faced). The constructed plasmid was introduced into *M. abscessus* strains by electroporation (BTXECM630, San Diego, CA, USA) at 2500 V, 1000 Ω, and 25 μF. 

### 2.4. Sliding Motility Assay

Sliding motility was examined as previously described [30]. *M. abscessus* cells were inoculated on 7H9 with 0.3% agar. After an incubation at 37 °C for 3 days, the sliding distance was measured.

### 2.5. Aggregation Assay

The aggregation assay was performed as previously described with modifications [22]. *M. abscessus* cells with an optical density of OD_600_ = 0.1 were cultured in a tube containing 5 mL of 7H9 medium with 10% OADC. After an incubation at 37 °C with continuous rotation for 2 days, the cultures were allowed to stand still at room temperature for 10 min. The upper portion of each culture containing dispersed cells was then removed to determine the OD_600_ value. The aggregated cells at the bottom of each tube were completely suspended by vortexing with glass beads of 4.5 mm in diameter (Biospec, Bartlesville, OK, USA), and the cell suspension was measured for its OD_600_ value. The aggregative index of each culture was calculated as the ratio of OD_600_ value of aggregated cells to that of dispersed cells [22].

### 2.6. Lipid Extraction and Thin-Layer Chromatography (TLC)

*M. abscessus* lipid was extracted as previously described [25,28,31]. *M. abscessus* cells were cultured for 4–5 days on agar plates, harvested, and incubated in 1 mL of chloroform/methanol (2:1, *v/v*) at 56 °C for 2 h in a water bath sonicator (Branson 5200). After centrifugation (14,000 rpm, 15 min), the supernatant (500 μL) was purified by extracting with 500 μL chloroform/methanol (2:1) and then with 1000 μL distilled water. The organic extracts were dried, weighted, and resuspended in chloroform/methanol (2:1). For saponification, 1:1 volume of 0.2 N NaOH and resuspended total lipid were mixed, incubated at 37 °C for 45 min, and then neutralized with glacial acetic acid (final concentration = 0.1 M). The saponified lipid was dissolved in chloroform/methanol, spotted on an aluminum-backed silica gel_60_ TLC plate (MERCK, Darmstadt, Germany), and resolved with 10 mL of chloroform/methanol/H_2_O (100:14:0.8). The TLC plate was soaked briefly in 10% H_2_SO_4_ in methanol and then heated to visualize lipids.

### 2.7. Intracellular Survival Assay

This assay was performed as previously described [28]. The human acute monocytic leukemia cell line THP-1 was acquired from ATCC. THP-1 cells were cultured in RPMI 1640 medium supplemented with 10% fetal bovine serum (GIBCO, Gland island, NY, USA) and differentiated into macrophage by treatment with phorbol-12-myristate-13-acetate (500 ng/mL) (Sigma, St. Louis, MO, USA) at 37 °C in a CO_2_ incubator overnight in 24 well plates (SPL Lifesciences) with a total amount of 500 μL, 2 × 10^5^ cells per well. The culture medium was refreshed, and the culture was incubated for 2 more days. The cells were then infected with *M. abscessus* at a multiplicity of infection (MOI) of 1 for 2, 24, 48, or 72 h. The infected cells were washed to remove extracellular bacteria. The washed cells were then lysed by incubation with 1% Triton X-100 for 30 min, and the cell lysate was plated on 7H11 agar plates to determine the number of intracellular mycobacteria [32,33].

### 2.8. Lysozyme and H_2_O_2_ Susceptibility Assay

*M. abscessus* cells were first cultured in 7H9 broth supplemented with 10% OADC and 0.5% Tween 80 at 37 °C for 3–4 days. Approximately 3 × 10^7^ of these cells were inoculated into 3 mL of 7H9 broth without OADC and with various concentrations of lysozyme (0, 0.5, 2.5 mg/mL) for 24 h or with H_2_O_2_ (0, 10, 20, 30 mM) for 2 h at 37 °C. The cultures were then serial diluted and plated (20 μL) on 7H11 agar plates for determination of bacterial CFU.

### 2.9. Sequence Alignment

According to the result of Kyoto Encyclopedia of Genes and Genomes (KEGG, http://www.genome.jp/kegg/ (accessed on 23 March 2021)) analysis, the functional role of Mab_3083c was recommended as RNase J. The following amino acid sequence alignments were performed by clustalW2 and NCBI program.

### 2.10. Statistical Analysis

The experimental data are presented as mean ± standard deviation. Statistical analysis was carried out by Student’s t-test, one-way ANOVA, and Tukey’s test for multiple comparisons.

## 3. Results

### 3.1. Characterization of the Mab_3083c::Tn Mutant

**Figure 1:**
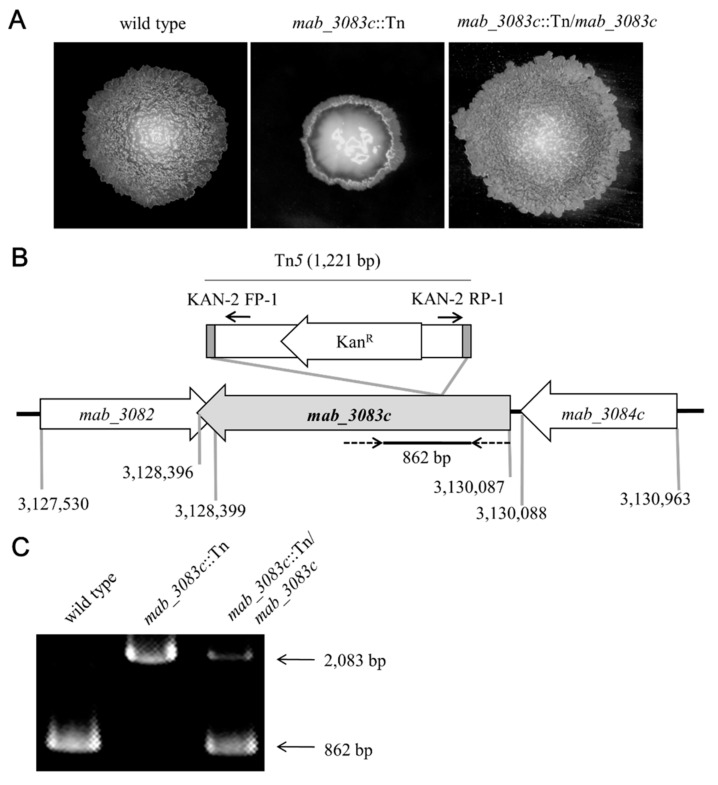
Characterization of mab_3083c transposon mutant. (**A**) Colony morphology of wild type, *mab_3083c*::Tn, and complemented (*mab_3083c*::Tn/*mab_3083c*) strains**.** Cells of each of these *M. abscessus* variants were grown on Middle brook 7H11 agar plate supplemented with 10% OADC and incubated at 37 °C for 5 days. (**B**) Diagrams of the *mab_3083c* locus and the inserted 1221-bp Tn*5* containing a kanamycin resistant gene (Kan^R^). Gray bars represent the terminal inverted repeats of Tn*5*. Numbers on the bottom of the figure are nucleotide positions of the *M. abscessus* genome (GenBank accession no. NC_010397). The transposon was inserted between nucleotides 365 and 366 downstream from the initiation codon of *mab_3083c*. Solid arrows(→)indicate primers KAN-2 FP-1 and KAN-2 RP-1 that were used to determine the insertion site of Tn*5*. Dotted arrows(-->)denote primers used to detect insertion of Tn5 into the *mab_3083c* gene. (**C**) Identification of Tn5 insertion into the *mab_3083c* gene. The wild type strain containing the intact *mab_3083c* gene yielded an 862-bp PCR product, while the *mab_3083c*::Tn mutant had a 2083-bp PCR product indicating insertion of Tn5 into the *mab_3083c* gene. The complemented strain (*mab_3083c*::Tn/*mab_3083c*) had both 862-bp and 2083-bp fragments, indicating that some copies of the *mab_3083c* gene from pYUB-*mab_3083c* were integrated into the chromosome of the strain.

To investigate factors associated with colony morphotype switching of *M. abscessus*, a transposon mutagenesis was performed on the rough strain cs1c-R (wild type). A mutant named *mab_3083c*::Tn with the colony morphotype switched from rough to smooth was obtained (Figure 1A, middle panel). To confirm that this morphotype switching was due to the defect in *mab_3083c*, the plasmid pYUB-*mab_3083c* containing the wild type *mab_3083c* of *M. abscessus* was introduced into the *mab_3083c*::Tn mutant. The colony morphology of this complemented strain (designated *mab_3083c*::Tn/*mab_3083c*) was found to revert back to rough (Figure 1A, right panel), indicating that *mab_3083c* is associated with the rough morphotype of *M. abscessus*.

To determine the insertion site of Tn*5*, genomic DNA was isolated from the *mab_3083c*::Tn mutant and then digested with BamHI. The resulting DNA fragments were circularized by ligation and used as template for PCR with primers KAN-2 FP-1 and KAN-2 RP-1 that anneal at the two ends of Tn*5* (Figure 1B). The PCR products thus obtained were sequenced. Results of this experiment showed that the Tn*5* was inserted into *mab_3083c* (GenBank accession no. NC_010397) between nucleotides 365 and 366 downstream from the initiation codon (Figure 1B).

To confirm the presence of *mab_3083c* in the genome of the complemented strain (*mab_3083c*::Tn/*mab_3083c*), PCR was performed with primers (Figure 1B, dotted arrow) designed to amplify a portion (862 bp) of *mab_3083c* (Figure 1B,C). As the control, the same PCR was performed on genomic DNA of the *mab_3083c*::Tn mutant, and a 2083-bp fragment (Figure 1C) was determined, indicating that the 1221-bp Tn*5* was inserted into the 862-bp fragment of *mab_3083c* (Figure 1B). Both the 2083-bp (Figure 1C) and 862-bp fragments (Figure 1C) were generated from the complemented strain (*mab_3083c*::Tn/*mab_3083c*), suggesting that some copies of the introduced *mab_3083c* on pYUB-*mab_3083c* were integrated into the chromosome of *mab_3083c*::Tn/*mab_3083c*.

### 3.2. No Association of GPL with Colony Morphotype Switching of the mab_3083c::Tn Mutant

**Figure 2:**
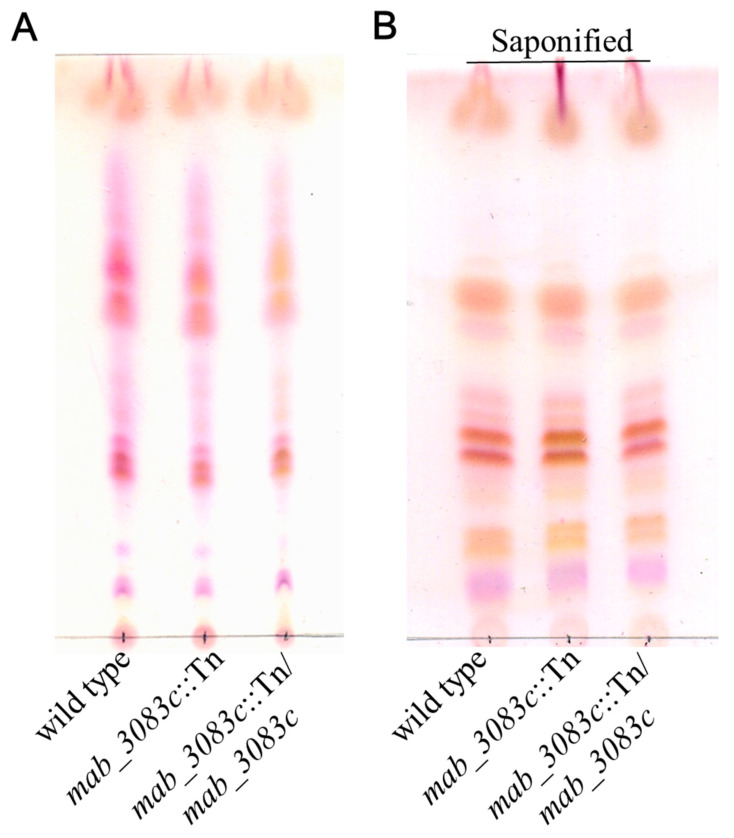
Comparison of glycopeptidolipid (GPL) production of wild type, mab_3083c::Tn, and complement strains of M. abscessus by Thin-Layer Chromatography (TLC). Total (**A**) and alkali stable (saponified) lipid (**B**) of wild type, *mab_3083c*::Tn, and complemented (*mab_3083c*::Tn/*mab_3083c*) strains visualized with H_2_SO_4_.

A previous study showed that the strain with a smooth colony morphotype contained higher amounts of GPL than the rough strain [21]. To investigate the GPL profiles of wild type (cs1c-R), *mab_3083c*::Tn, and complemented (*mab_3083c*::Tn/*mab_3083c*) strains, lipids of these strains were extracted and analyzed by TLC. The lipid profiles of three strains were no significant difference (Figure 2A,B).

### 3.3. No Association of Mab_3083c with Intracellular Survival and Susceptibility to Hydrogen Peroxide and Lysozyme of M. abscessus

**Figure 3:**
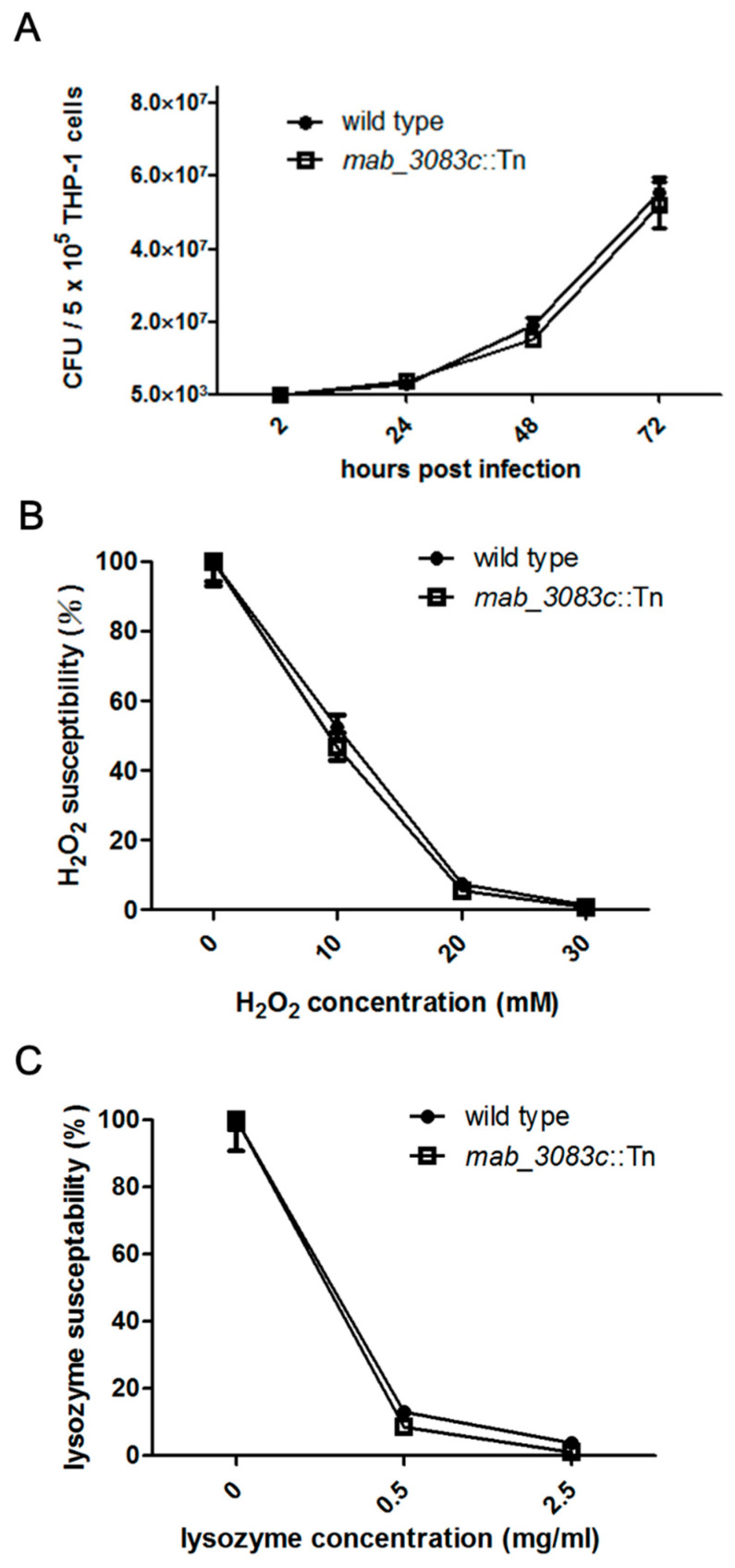
Intracellular survival and susceptibility to hydrogen dioxide and lysozyme of wild type and mab_3083c::Tn strains of *M. abscessus*. (**A**) Intracellular survival of *M. abscessus*. THP-1 cells were co-cultured with bacterial cells of wild type or *mab_3083c*::Tn mutant at MOI of 1 for 2, 24, 48, or 72 h at 37 °C. The infected THP-1 cells were washed and lysed, and the numbers of survived intracellular *M. abscessus* cells were determined at the indicated time points. (**B**) Susceptibility of *M. abscessus* to H_2_O_2_. Approximately 3 × 10^7^ bacteria were inoculated into 7H9 broth containing 10, 20, or 30 mM of H_2_O_2_ and incubated at 37 °C for 2 h. The CFU of the treated cells was then counted, and the susceptibility to H_2_O_2_ was determined. (**C**) Susceptibility of *M. abscessus* to lysozyme. Approximately 3 × 10^7^ bacteria were inoculated into 7H9 broth containing 0.5 or 2.5 mg/mL of lysozyme and incubated at 37 °C for 24 h. The CFU of the treated cells was counted, and the susceptibility to lysozyme was determined.

It has been shown that cells of the rough strain of *M. abscessus* can persist in human monocytes, while those of the smooth strain are rapidly cleared [20]. To investigate whether *mab_3083c* is associated with the intracellular survival of *M. abscessus*, THP-1 cells were respectively infected with the wild type (cs1c-R) and the *mab_3083c*::Tn mutant at an MOI of 1, and the CFU of intracellular bacteria was counted at 2, 24, 48, and 72 h post infection. As shown in Figure 3A, at 2 h post infection, the CFU of the mutant ((5.2 ± 0.5) × 10^3^) was similar to that of the wild type ((3.7 ± 0.6) × 10^3^). No significant difference was observed at 24, 48, and 72 h post infection between the wild type ((2.8 ± 0.5) × 10^5^ at 24 h; (1.9 ± 0.5) × 10^7^ at 48 h; (5.6 ± 1.0) × 10^7^ at 72 h) and the mutant ((3.6 ± 0.2) × 10^5^ at 24 h; (1.5 ± 0.3) × 10^7^ at 48 h; (5.2 ± 1.5) × 10^7^ at 72 h).

To investigate the susceptibility of the wild type and the *mab_3083c*::Tn mutant to oxidative stress and lysozyme, the bacteria were incubated with 10–30 mM hydrogen peroxide for 2 h or 0.5–2.5 mg/mL lysozyme for 24 h. Bacteria incubated with broth without hydrogen peroxide or lysozyme were used as controls. The susceptibility was expressed as the percentage of CFU of bacteria with treatment divided by that without treatment. The results showed that the percentages of susceptibility of the wild type and the mutant were 52.3 ± 8.7 and 46.8 ± 9.4 to 10 mM H_2_O_2_, 7.4 ± 0.8 and 5.6 ± 0.7 to 20 mM H_2_O_2_, and 1.4 ± 0.4, and 0.9 ± 0.3 to 30 mM H_2_O_2_, respectively (Figure 3B). The percentages of susceptibility of the wild type and the mutant were 13.0 ± 1.4 and 8.6 ± 0.7 to 0.5 mg/mL lysozyme, 3.9 ± 0.6 and 1.0 ± 0.5 to 2.5 mg/mL lysozyme, respectively (Figure 3C). Since there was no significant difference in H_2_O_2_ and lysozyme susceptibility between the wild type and the mutant, it is unlikely that *mab_3083c* regulates the intracellular survival of *M. abscessus*.

### 3.4. Higher Sliding Motility and Less Aggregation Capability of the Mab_3083c::Tn Mutant

**Figure 4:**
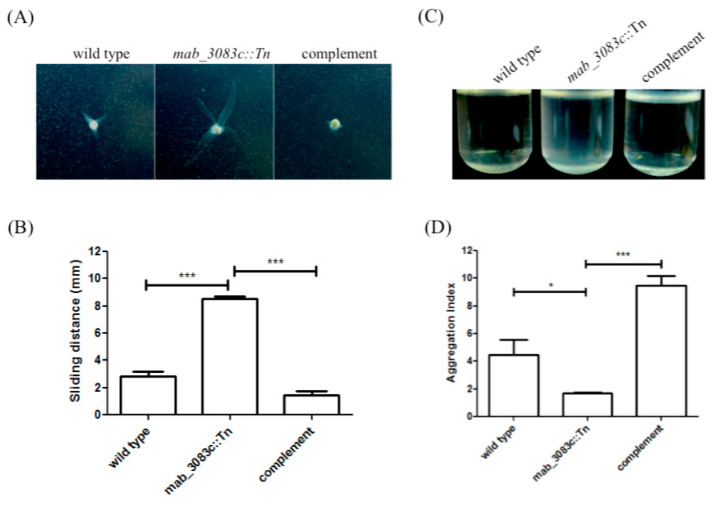
Sliding motility and aggregation capability of *M. abscessus* wild type, mab_3083c::Tn, and complemented strains. (**A**) Sliding ability of the tested strains. (**B**) Quantification of sliding ability. The sliding distance was measured in mm and plotted. (**C**) Aggregation capability of the tested strains. (**D**) The aggregation index of these three strains. Data were analyzed with one-way ANOVA and Tukey’s multiple comparison. * *p* < 0.05, *** *p* < 0.001.

According to previous studies, smooth strains of *M. abscessus* displayed higher sliding motility and less aggregation capability [22,30]. To test whether the *mab_3083c*::Tn mutant have similar properties, its motility was examined on 7H9 medium with 0.3% agar. The sliding distance was measured and plotted (Figure 4A,B). The wild type strain was found to have a low motility (2.83 ± 0.38 mm), while the *mab_3083c*::Tn mutant was highly motile (8.56 ± 0.18 mm). The *mab_3083c* complemented strain (*mab_3083c*::Tn/*mab_3083c*) was similar to the wild type with a low sliding phenotype (1.44 ± 0.29 mm).

To test whether *mab_3083c* affects the aggregation capability of *M. abscessus*, the variants were grown in 7H9 media with 10% OADC and 0.05% Tween 80 for two days. Cultures of the *mab_3083c*::Tn mutant were found to be turbid, whereas those of the wild type and the complemented (*mab_3083c*::Tn/*mab_3083c*) strains had bacterial aggregates at the bottom and clear supernatants. To quantify the aggregation ability, the aggregation index of each culture was calculated and plotted. As shown in Figure 4C,D, the *mab_3083c*::Tn mutant (1.70 ± 0.03) displayed a lower aggregation ability than the wild type (4.49 ± 0.62), and the *mab_3083c* complemented strain (9.49 ± 0.69) regained the aggregative phenotype. These results suggest that *mab_3083c* is associated with a low sliding and a high aggregation ability of *M. abscessus.*

### 3.5. Identification of Mab_3083c as a Homologue of Ribonuclease J

**Figure 5:**
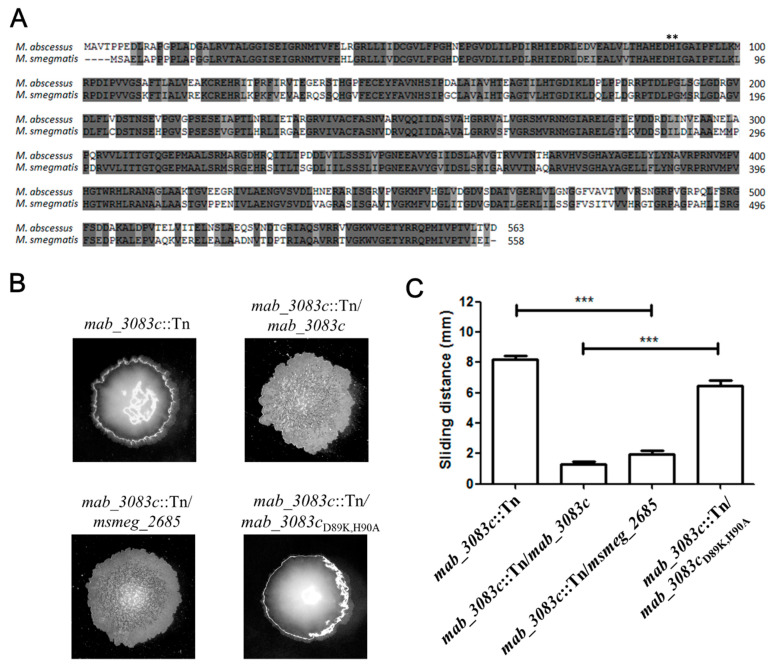
Identification of *mab_3083c* (**A**) Alignment of RNase J amino acid sequences of *M. abscessus* and *M. smegmatis*. Dark gray and light gray represent same and similar amino acid, respectively. Stars represent the mutation sites (*mab_3083c*, D89, and H90). Sequences were downloaded from NCBI (*M. abscessus* ID: NC_010397.1; *M. smegmatis* ID: NC_008596.1) and aligned using Vector NTI alignment X (Invitrogen, Frederick, MD, USA). Colony morphology (**B**) and sliding ability (**C**) of *mab_3083c*::Tn, *mab_3083c*::Tn/*mab_3083c*, *mab_3083c*::Tn/*msmeg_*2685, and *mab_3083c*::Tn/*mab_3083c*_D89K, H90A_ strains. Statistical analysis was performed by one-way ANOVA and Tukey’s multiple comparison tests. *** *p* < 0.001.

Amino acid sequence alignment between Mab_3083c and RNase J of *M. smegmatis* mc^2^155 (*msmeg_2685*) indicated that they share a 75.4% identity (Figure 5A). To confirm that Mab_3083c possesses RNase J activity, the *msmeg_2685* gene, which encodes RNase J of *M. smegmatis*, was introduced into the *mab_3083c*::Tn mutant by electroporation of pYUB-*msmeg_2685*. The resulting strain was designated *mab_3083c*::Tn/*msmeg_2685*. This *msmeg_2685* complementation was found to revert the colony morphology of the *mab_3083c*::Tn mutant back to the rough morphotype (Figure 5B), the same as complementation with *mab_3083c* (Figure 1A and Figure 5B).

It has been shown that D85K and H86A mutations generated at the active site of *M. smegmatis* RNase J resulted in loss of its RNase activity [34]. To prove that Mab_3083c has RNase J activity, similar mutations, D89K and H90A, were generated in *mab_3083c* on pYUB-*mab_3083c.* The resulting plasmid was named pYUB-*mab_3083c*_D89K, H90A_ and introduced into the *mab_3083c*::Tn mutant to perform the complementation experiment. The new variant designated *mab_3083c*::Tn/*mab_3083c*_D89K, H90A_ was found to have a smooth morphotype (Figure 5B) and higher sliding ability (Figure 5C), the same as the *mab_3083c*::Tn mutant, indicating that the mutated *mab_3083c* gene (*mab_3083c*_D89K, H90A_) failed to complement the *mab_3083c*::Tn mutation. Taken together, these results provided evidence that Mab_3083c functions as an RNase J.

## 4. Discussion

GPLs are a member of glycolipids produced by many mycobacteria genus involved in morphotype switching, biofilm formation, sliding motility, pathogenicity, and immunomodulation [26,35,36,37]. Notably, many studies demonstrated that absence of GPL in *Mycobacterium* promotes bacterial aggregation and loses the ability to produce biofilm and sliding ability [26]. In this study, morphotype switching strain (*mab_3083c*::Tn mutant) was selected. Interestingly, no significant differences were found between wild type and mutant in GPL profiles, intracellular survivability, susceptibility to hydrogen peroxide and lysozyme. According to bioinformatics analysis, the role of *mab_3083C* was recommended as RNase J. Phenotype alterations regulated by RNase J have been reported in *Bacillus subtilis* [38] and *Streptomyces venezuelae* [39]. RNase J1 knockout in *Bacillus subtilis* affected its spore formation and maturation as well as alteration in cell appearance with disordered peptidoglycan layer and long spiral filaments [38]. The RNase J mutant of *Streptomyces venezuelae* also has defects in cell development, sporulation, and ribosome assembly. In our study, the RNase J mutant of *M. abscessus* (*mab_3083c*::Tn) was viable, suggesting that RNase J is not essential for its growth, unlike *Streptococcus pyogenes* in which both RNase J1 and RNase J2 are essential [40].

As some ribonucleases, RNase J regulates mRNA stability or ribosomal RNA maturation [34,41,42,43]. In Gram-positive bacteria, RNase J is functionally equivalent to RNase E, which plays an important role in mRNA decay in Gram-negative bacteria [44]. According to the structure of *Thermus thermophilus* RNase J [45], RNase J possesses three domains: β-lactamase domain, β-CASP domain, and C-terminal domain. D85 and H86 in RNase J_Msmeg_ are located at the active site of the β-lactamase domain, and D85K and H86A mutations of *M. smegmatis* RNase J result in the loss of its exonuclease activity [34].

Some studies have described the role of RNase J in mRNA turnover. For example, the RNase J1/J2 double mutant of *Bacillus subtilis* was found to have increased levels of approximately 300 mRNA transcripts and decreased levels of equal numbers of mRNA transcripts [46]. Studies have also shown that RNase J can affect the stability of specific mRNAs [44,46]. It is possible that the RNase J (*mab_3083c*) of *M. abscessus* affects the stability of some mRNAs related to its colony morphology, sliding motility, and aggregation ability. Further studies are warranted to identify the genes encoding these mRNAs.

## 5. Conclusions

Most reports on the morphotypic switch of *M. abscessus* were associated with GPL locus. Our study demonstrated that the non-GPL locus gene *mab_3083c* also plays a role in this switch. The disruption of *mab_3083c* led to colony morphology switching from rough to smooth, increased sliding motility, and reduced aggregation ability of *M. abscessus*. Complementation of the *mab_3083c*::Tn mutant with RNase J_Msmeg_ (*msmeg_2685*) switched its colony morphology from smooth back to rough. These results suggest that *mab_3083c* is the RNase J gene of *M. abscessus*.
